# Nutrition, Environment, and Genetics in Colorectal Cancer. Epigenetics and Possible Future Perspective

**DOI:** 10.1007/s13668-025-00701-9

**Published:** 2025-10-18

**Authors:** Stefano Brandolino, Marica Franzago, Giovanna Murmura, Fabrizio Ricci, Valentina Gatta, Liborio Stuppia, Ester Vitacolonna

**Affiliations:** 1 Department of Medicine and Aging, “G. D’Annunzio” University, Via Dei Vestini, Chieti-Pescara, 66100 Chieti, Italy; 2 Center for Advanced Studies and Technology (CAST), “G. D’Annunzio” University of Chieti-Pescara, 66100 Chieti, Italy; 3 Department of Innovative Technologies in Medicine and Dentistry, “G. D’Annunzio” University of Chieti-Pescara, 66100 Chieti, Italy; 4 Department of Neuroscience, Imaging and Clinical Sciences, “G. D’Annunzio” University of Chieti-Pescara, 66100 Chieti, Italy

**Keywords:** One health, Diet, Precision nutrition, Environment, Nutrigenomics, Inflammatory bowel disease

## Abstract

**Purpose of Review:**

This review provides an overview of the relationship among nutritional, environmental, and genetic factors in the development of the chronic inflammatory state, starting from the inflammatory bowel disease (IBD) up to the onset of Colorectal Cancer (CRC). Finally, it also examines potential prospects and future topics of research taking into account the relation between nutrition and epigenetic factors.

**Recent Findings:**

Evidence indicates that genetic and lifestyle-related factors play a crucial role in CRC etiology. Dietary intake may induce epigenetic alterations which in turn, result in carcinogenesis. Several bioactive components can modify epigenetic mechanisms, required for gene activation or silencing, thus, representing a potential way of preventing CRC.

**Summary:**

The gene-diet interaction analysis suggested some functions and pathways that may affect the CRC development. In this view, personalized nutrition, which is an approach that combines with new omics technologies, could represent a new possible key for personalized prevention and treatment in association with other cancer prevention and chemotherapeutic therapies.

## Introduction

Colorectal cancer (CRC) is the third most prevalent cancer globally [1], the leading cause of cancer death first in men and second in women [2, 3]. After the diagnosis, the survival index after 5 years is around 60% [4]. The stage at the diagnosis is strictly linked to the survival rate, it’s higher for localized disease but much lower for distant disease [5]. Previous studies have reported an association between the risk of CRC and several lifestyle-related factors including obesity [6], alcohol and processed meat consumption [7], smoking [8], and dysbiosis [9]. Among the risk factors, a history of inflammatory bowel disease (IBD) can increase the possible onset of Early-onset colorectal cancer (EoCRC). Up to three times more than people with Later onset CRC (LoCRC) and, the incidence of EoCRC in the population with IBD is sixfold higher [10]. IBD, which is characterized by the interaction among altered microbiota as well as genetic and environmental factors, comprises two main subtypes including Crohn’s disease (CD) and Ulcerative colitis (UC). It is estimated that 7 million people suffer from IBD, which arises following incompletely defined environmental triggers in genetically predisposed subjects [11]. These conditions are characterized by an inflammation of the digestive tract as well as dysfunction of the immunological reaction alternating between recurrence and remission phases [12].

IBD lasts for the whole life, and to date, a medication or treatment, which can completely stop the disease, have not yet been identified [13]. Immunosuppressive, anti-inflammatory, biological, and immunomodulatory drugs are widely used, however dietary interventions have been less considered [12]. Patients with IBD have an increased risk of developing colitis-associated CRC, with an incidence of approximately 5% after 20 years of disease. As a result, they are enrolled in an endoscopic surveillance program to identify early signs of cancer in a timely manner [14]. From perspective genetic profiling, there are differences in the pathogenesis of CRC.

Clarifying the genetic diversity between colitis-associated CRC and sporadic-CRC is crucial for clinical management [15, 16]. Albeit molecular differences are not completely understood, mutations of the key driver genes like *APC* and *KRAS* are less prevalent in colitis-associated CRC whereas the *TP53* mutation shows up earlier in colitis-associated CRC compared to sporadic counterparts [14]. A study on whole -exome sequencing demonstrated distinct genetic features including specific gene mutations (such as *SOX9*, *EP300, NRG1*, and *IL16*) in tumors from IBD patients compared to sporadic colorectal tumors [17]. Moreover, Baker et al. [18] confirmed that colitis-associated CRC shows a modest rise of single nucleotide alterations including repeated mutations in genes infrequently mutated in sporadic-CRC. This finding has been supported by a meta-analysis conducted by Li et al. [19], identifying several single nucleotide polymorphisms (SNPs) that were significantly associated with the risk of IBD-associated CRC.

On the epigenetic side, chronic inflammation is also associated with aberrant DNA methylation of several genes involved in cell-cycle regulation, aging, and, tumor suppression like *RUNX3*, *MINT1*, and *COX-2* linked to ulcerative colitis -associated CRC in non-neoplastic tissue. Moreover, the regulation of specific micro RNAs (miRNAs), noncoding RNAs, is linked to cellular homeostasis [20].

The molecular mechanisms linking IBD, CRC, and nutrients are not yet established. IBD patients are struggling to keep an adequate nutritional intake and a balanced diet due to digestion, absorption of nutrients, and appetite changes. These factors could lead to an alteration to several signaling pathways, such as mTORC1 pathway, potentially inducing DNA damage and triggers chromosomal instability in IBD, thus increasing CRC risk [21]. Future studies could develop optimal personalized therapy for CRC based on these findings.

The above-described aspects suggest the important role of genetic factors in cancer development, and furthermore, DNA instability and gene alterations can be affected by diet. In this view, dietary intake and nutrient supplements may also induce epigenetic alterations which in turn, results in carcinogenesis. These aspects led to the introduction of the concept of nutrigenomics and nutrigenetics which may explain the relation between specific nutrient intake with genetic variants on cancer pathogenesis. This review provides an overview of the actual knowledge regarding the relationship between the diet, with a particular focus on the genetic and environmental aspects, and the chronic inflammatory state, starting from the IBD up to the onset of CRC. We also examine potential prospects and future topics of research taking into account the relationship between nutrition and epigenetic factors.

## Nutrition, Inflammation, and CRC

The malnutrition, obesity, and diabetes are relevant risk factors for IBD and CRC [22]. It’s well known that specific dietary patterns play a fundamental role in the prevention of several diseases. The low animal protein content, plus a low glycemic index and the high levels of nutraceutical and bioactive compounds give an anti-inflammatory activity to the Mediterranean Diet [23].

Conversely, pro-inflammatory activity of different types of diet, e.g. Western Diet, which is characterized by high consumption of animal fats and proteins, refined sugars, and reduced intakes of fruit, vegetables, and fiber, can trigger diseases like IBD and Cancer, due to its negative influence on chronic intestinal inflammation and oxidative stress [24]. Thus, a typical Western diet pattern, with high consumption of refined carbs and sugar is characterized by a high glycemic load, which is related to hyperinsulinemia and accordingly related to CRC increased risk. Furthermore, for some macronutrient groups, e.g. carbohydrates, the choice of food category could be a protective or harmful factor; indeed, it has been shown a positive association between CRC and high intake of monosaccharides\ disaccharides glucose, fructose, maltose and sucrose [25].

The Western diet is also rich in ultra-processed food (UPFs) that contains lots of food additives (e.g. emulsifiers); these components are used to improve food's texture, palatability, and shelf life. Thus, the emulsifiers have been associated with intestinal inflammation and the onset of IBD due to their action on permeability of epithelial cells and promotion of the secretion of inflammatory cytokines, like the Tumor necrosis factor (TNF) [26].

Patients with chronic inflammation in gastrointestinal tissue have a major risk of neoplasia. Moreover, factors like the extent, duration, and grade of dysplastic lesions are directly linked to the onset of colitis-associated CRC; hence, generally: i) subjects with chronic intestinal inflammation and IBD, the onset of CRC is diagnosed early; ii) is characterized by genomic instability; iii) the risk increase at the rate of 0.5 to 1% per year compared to sporadic colorectal cancer in the general population [27].

Another factor directly linked to intestinal inflammation and the development of IBD and CRC is the gut microbiota, and nutrition represents one of the main factors affecting its composition and variability [24]. Indeed, the incidence of IBD is higher in developed countries, in relationship with the typical lifestyle and the so-called “Western Diet” that reduce the diversity of the bacterial in the bowel triggering the leaky gut syndrome and the inflammatory process [28].

**Fig. 1:**
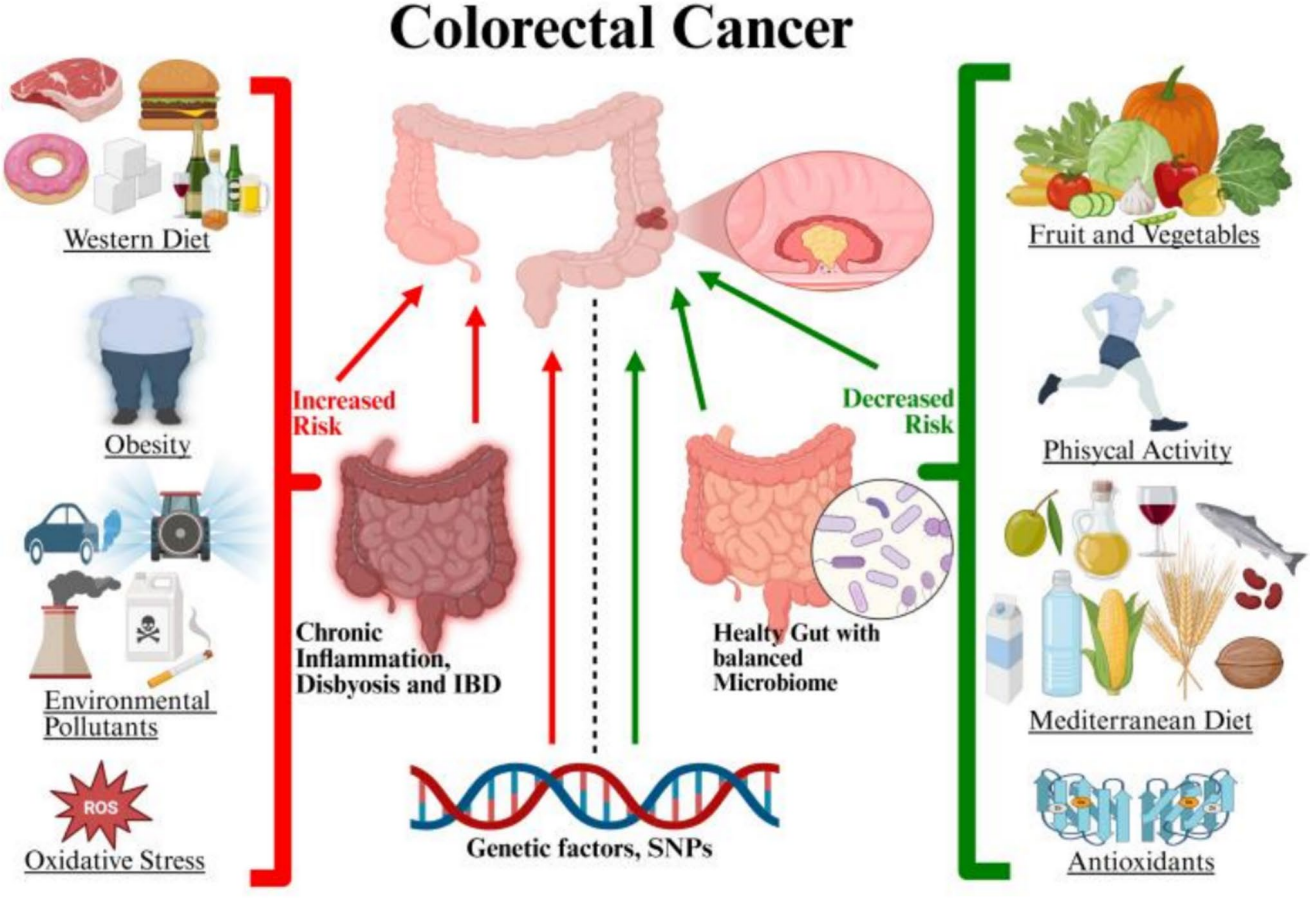
Triggers and protective factors potentially influencing the gut’s health. An unhealthy lifestyle, environmental pollutants, and oxidative stress can lead to chronic inflammation, dysbiosis, and Inflammatory Bowel Diseases (IBD) that are related to Colorectal Cancer (CRC). On the other hand, a healthy lifestyle, with a balanced diet rich in fruit and vegetables as well as physical activity, can play a positive role in preventing CRC. Furthermore, genetic factors, like SNPs, can influence this process. The original figure was created with BioRender.com

However, a diet rich in vegetables and fiber has been related to an enrichment and higher diversity of the microbiome and this is directly linked to a reduction in the risk of the onset and progression of IBD and CRC [24] (Fig. 1).

The potential role of single micro and macronutrients in CRC remains controversial. An Italian case–control study, including 1,225 CRC subjects, identified 5 major dietary patterns, showing the Starch-rich pattern potentially an unfavorable risk predisposing to both colon and rectal cancer, whereas the unsaturated fats patterns associated with a reduced risk of colon cancer [29].

Some studies support the beneficial role of Ca ^+2^ and vitamin D supplementation in tumor suppression, by regulating proliferation, apoptosis, inflammation pathways [30–32]. However, other studies, based on the use of Ca ^+2^ and vitamin D, have not confirmed a significant reduction in incidence [33, 34]. Recently, the study conducted by Wang et al. [35] is the first to perform a systematic analysis of vitamin D levels and related genes, identifying five hub genes, including *SOSTDC1*, *CCND1*, *CEACAM1*, *MMP1*, and *PRKAA2*, as potential novel biomarkers for CRC. These authors showed that vitamin D intervention can modulate these genes’ expression, highlighting its role in CRC development. Vitamin A and E are also known for their anti-oxidative and anti-inflammatory effects [31] and clinical trials have been showed a reduced risk of tumor development upon vitamin supplementation [36]. Further research is still required to elucidate the potential contribution of these vitamins in tumor progression and to identify the population that may benefit by individual treatment [22]. Several dietary constituents can work on an epigenetic level through the production of epigenetic substrates or regulators of chromatin-modifying enzymes, and therefore they directly can lead to the aberrant expression of genes involved in immune response, and intestinal barrier function, resulting in chronic inflammation. Hence, the diet, through these mechanisms can influence the symptoms, flare up and the overall management and outcome of the IBD [28].

Folate (B9), riboflavin (B2), pyridoxine (B6), cobalamin (B12) are involved in DNA methylation, synthesis, stability, and repair [31], with beneficial role in intestinal homeostasis.

These methyl donor nutrients could affect DNA methylation, restoring proper gene expression patterns and improving the IBD symptoms. However, as highlighted by Marangoni et al. [28], further research is need to provide the optimal doses and the period of dietary exposure or depletion that induces modifications in epigenetic marks. In addition, evidence suggests that the epigenetic modulation also induced by omega-3 fatty acids, vitamin A, SCFAs and polyphenols can reduce inflammation in IBD patients, on the other hand it is important to underline that more studies in this context are needed to understand the mechanisms, the dosages and effects for using these nutrients in managing IBD [28].

## Environmental Exposure, Inflammatory Bowel Diseases, and CRC

Germline mutations cause 10–15% of CRC cases whereas environmental insults account for 80–90% [37]. It is well known that CRC is a multifactorial disease influenced by internal factors (inflammation, dynamics of gut microbiota, genetics, and age) [38–43], and external exposures (diet, smoking, drinking, industrial pollution) [44–46]. As a matter of fact, looking at epidemiological data, seems that the surge of cases of EoCRC is not related to genetic evolution, but they are initiated and enhanced by environmental factors [47]. People born after 1950 seem at major risk of developing EoCRC from increasing exposure to chemicals like pesticides and pollution [37]. For instance, the International Agency for Research on Cancer (IARC) has classified outdoor air pollution as a Group 1 carcinogen; linking it to lung cancer and cancers of other sites, including the colon-rectum [48]. This correlation with CRC incidence and survival may be influenced by epigenetic factors, mainly methylation of specific protein coding genes, which can be responsible for the pathogenic effect of air pollution on CRC [49]. Other potential connections between environmental factors and this specific cancer are represented by microplastics (MPs). Since 1950, exposure to these substances has surged alongside the increased production and use of plastics. In the gastrointestinal tract, MPs can reach the colon, where they damage and disrupt the mucus layer, resulting in damage to the intestinal barrier. Consequently, this diminishes its protective role, which may contribute to EoCRC development [50]. Also low-dose of Bisphenol A (BPA), commonly used in polycarbonate plastics and epoxy resins, can affect cancer progression by several molecular signaling pathway [51, 52] such as ceramide metabolism [53]. Moreover, among worrying factors linked to the EoCRC, there are also Polycyclic aromatic hydrocarbons (PAHs); these substances include over 200 organic compounds which can enter the human body mainly through the gastrointestinal tract, because they are largely collected in the alimentary chain due to their lipophilic propriety. Cooked protein products, especially grilled red meat, are one of the main sources of PAHs [54]. Thus, the unhealthy eating patterns, very common especially among the younger generation, are characterized by several eating habits, including an increased consumption of processed and barbecued meats, potentially linked to rising EoCRC cases [55]. PAHs in meat arise from nutrient pyrolysis and smoke from incomplete combustion; the body metabolizes these substances into compounds that can damage DNA in the colon-rectum, raising the risk of cancer [56], especially in younger individuals, due to early exposure and raised ingestion of meat and grilled food, as mentioned before. Furthermore, road traffic is recognized as a significant primary contributor to PAHs emissions in urban areas. Estimates suggest that emissions from motor vehicles are responsible for approximately 46% to 90% of total PAHs levels found in ambient particulate matter (PM) [57]. Indeed, PAHs, which have been related to the onset of CRC in several studies, have also been detected in widely consumed leafy vegetables, which were heavily contaminated with these compounds [58].

Then, growing environmental exposure mainly results from rural-to-urban migration, leading to lifestyle changes (including eating habits) and increased pollution. Moreover, since 1950 many new pesticides have been synthesized and their effect on human health is yet unknown. Environmental insults impair health through genomic alterations and mutations, mitochondrial dysfunction, altered intercellular communication, and epigenetic alterations. The first organs that can be marked by these exogenous insults are the organ barriers, including the microbiome [59]. These substances have the potential to interfere with the composition and homeostasis of the gut microbiota, triggering dysbiosis, chronic inflammation, and oxidative stress, affecting the host’s immune response, factors that overall amplify the risk of EoCRC [37].

Recently, it has been introduced the exposome concept which is related to a range of exposures including ecosystem, lifestyle, social, and physical–chemical domains, as well as their corresponding biological responses [60]. Nowadays, the evaluation of the human exposome is advancing with interest, since environmental factors can significantly affect the global burden of disease. On the other hand, it is very difficult to capture the influence of all exposome insults on health outcomes and to date, the studies based on the application of the exposome approach in the risks of CRC are lacking. Recently, Chen et al. [56] suggest that three exposure domains (ecosystem, lifestyle, and social factors) can be incorporated into prediction models to identify subjects at high risk of CRC. Therefore, the exposome, which reflects non-genetic factors, have the potential to impact on the development of disease, however future research directions in the fields are need.

## Obesity, Inflammation, Genetic Factors, and CRC

It’s well established that overweight (BMI > 25) and obesity (BMI > 30) are linked to an increasing risk of several kinds of cancers related to chronic inflammation and hypoxia, thus resulting in neovascularization [61]. About 30–70% of CRC has been correlated with obesity and abdominal fat storage [62]. In addition, each increase of 5 kg/m^₂^ in BMI is associated with a 17% higher risk of developing CRC [63, 64]. Obesity and CRC are directly linked: on one side, obese subjects are more likely to develop EoCRC [10]; on the other side, early-life obesity is associated with a major rate of CRC in adulthood. Despite that, the relationship between obesity and CRC and its mechanism of action is not fully clear [62].

One of the main factors that characterize obese patients is low-grade systemic inflammation; indeed, this condition that occurs for a long time may explain the connection between cancer and obesity. Thus, the interaction between infiltrating immune cells and adipocytes stimulates the pro-inflammatory cytokines production [10], which mainly are Interleukin-6 (IL-6), Tumor Necrosis Factor-alpha (TNFά) and Metalloproteinase-9 (MMP-9). In addition, the fat cells generate the Cytokine monocyte chemoattractant protein-1 (MCP-1) promoting tumorigenesis through inflammation. Other factors, like an increasing secretion of insulin and IGF factor, adipokines dysregulation, and an enhanced level of estrogens may contribute to obesity-related CRC development [62].

Another factor that characterizes individuals with obesity is an unbalanced and altered gut microbial population which could be linked to the onset of CRC; indeed, the connections between obesity-inducted dysbiosis and the onset of CRC are various, among which: i) a direct link between dysbiosis low-grade chronic inflammation which enhances colorectal tumorigenesis; ii) an altered production of metabolites, many of which are toxic and carcinogenic; iii) a dysfunction in energy metabolism (e.g. insulin resistance) that promote the onset of obesity associated CRC [65].

In this view, patients should be encouraged to weight loss and regular physical activity to prevent CRC incidence or reduce mortality in obese subjects who have already CRC. In addition, it has been suggest that obese subjects should be screened for CRC using BMI and waist circumference and counseled related to the importance of weight reduction, diet, and exercise [62]. There is also a genetic correlation between BMI and CRC risk. Moreover, fat mass and obesity associated (*FTO*) gene, is strongly linked to body weight and obesity [66–68]. It has been reported that the AMP-activated protein kinase (AMPK) activity influences *FTO* gene expression playing an important role in cell growth and proliferation through protein kinase B (AKT) and the mammalian target of rapamycin (mTOR) signaling pathway [69]. Although FTO has emerged as a potential biomarker in cancer research due to its critical function in RNA epigenetics, particularly in regulating m6A demethylation [70, 71], the role of *FTO* in CRC is not yet clear.

To date, GWAS has identified approximately 200 SNPs associated with CRC risk. Aglago et al. [63], recently identified rs58349661 within the *FMN1*/*GREM1* gene region, that interacts with the BMI in relationship with CRC risk. Particularly, overweight or obese individuals with *CC* and *CT* genotypes of this genetic variant, showed a higher risk of developing colorectal cancer compared to those with the *TT* genotype [63].

Thus, considering the relationship between the two complex diseases, it will be necessary to improve the management of obesity and its related care strategies to reduce the risk of CRC in this specific population.

This could be done through the integration of Nutrigenomics and Nutrigenetics approaches due to their consideration of two main aspects: genetic predisposition and gene expression changes related to diet. Therefore, the aim can be achieved by utilizing an extensive method that includes the evaluation of polymorphisms impacting energy metabolism and body composition together with the analysis of epigenetic processes [72].

## Nutritional Genomics

The Gene-Diet interaction can be impacted by several components including: i) Exposure (Intake of a different and new nutrient in a specific population); ii) Genomic (Individual or Ethnic/racial groups within a population with specific genetic variants that confer a different ability to metabolize a particular nutrient); iii) Epigenomic (Epigenetic changes that influence gene expression and the metabolism of nutrients) [73–77].

More specifically, nutritional genomics, which explores the interaction between nutrients and genes, includes Nutrigenetics and Nutrigenomics. These new fields of genetic science will help to identify the heritability of the genetic variants related to complex diseases [73].

Even though Nutrigenetics and Nutrigenomics are based on different scientific approaches, these two disciplines could be considered together; indeed, certain dietary molecules can potentially modify cellular homeostasis but the alteration of the homeostatic mechanisms occurs more in individuals with susceptible genotypes [78]. Nutrigenetics explores the interaction between the human genome and nutrition, determining how individual genetic profile and metabolic responses influence health status and disease susceptibility [79].

Through the technological advances, some studies have shown that gene-diet interactions, assessed across the genome, influence the CRC risk [80, 81], suggesting that dietary modifications could play a role in CRC prevention. In this view, Hoang et al. [80] demonstrated that individuals who carry multiple variants of *EPDR1* gene showed a correlation between fish intake more than 2 times per week and reduction of CRC risk. The EPDR1, which is upregulated in CRC patients, is involved in cell proliferation and migration, interaction with type I collagen fibrils, metabolism, and invasiveness in CRC cell lines [82, 83]. Moreover, several sets of protein-coding genes, which were overrepresented with particular pathways, interacted with the consumption of milk (ART), cheese (OR), tea (KRT), and alcohol (PRM and TNP) [80]. The effect of dietary flavonoid intake on CRC risk differs according to flavonoid subclasses and SNPs in *CYP1A1* gene. In particular, carriers of the *CYP1A1* rs4646903 *CC* with higher intake of flavonols and flavan-3-ols showed a decreased risk of CRC [84]. Based on this finding, dietary flavonoids and genetic variants, influencing CYP1A1 activity might impact carcinogen metabolism [85]. On the other hand, Nutrigenomics is a science of nutrition that seeks how a specific type of diet leads to several responses among different groups of individuals or populations via epigenetic mechanisms [86].

Epigenetics is defined as the study of the molecular mechanisms that establish and maintain mitotically stable patterns of gene expression yet do not affect DNA sequence [87]. These mechanisms (including DNA methylation, histone modifications and miRNAs), which regulate several processes, can be affected by the signal from the environment, thus influencing the phenotype [88]. The study of the epigenetic mechanisms, i.e. the interaction between the foods, their nutrients as well as bioactive compounds, and the human body can help in part to clarify the missing heritability problem of the GWAS studies that can’t fully explain the heritability of complex traits; in fact only a slight percentage, around 5–10%, of the genetic variants can explain the phenotype's heritability [77].

The rising interest in cancer epigenetics is related to the fact that alterations in epigenetic processes are involved in cell proliferation, differentiation, DNA repair and survival and thus, implicated in some steps of tumorigenesis [89].

In this view, Nutrigenomics and Nutrigenetics could elucidate how specific nutrient intake is associated with genetic variations in the pathogenesis of cancer [90].

More interestingly, as summarized in a study of Franzago et al. [75], the dynamic factors of the epigenome suggest that some epigenetic programming events can also be reversible. Considering this, many bioactive dietary components have been identified which can actively interfere with epigenetic targets. As covered in the next section, from a nutrigenomics point of view, several bioactive components including curcumin, genistein, tea polyphenols, resveratrol, and sulforaphane (cruciferous vegetables) can modify epigenetic mechanisms, particularly the DNA methylation and histone modifications, required for gene activation or silencing not only in the prevention but also in the therapy of a wide variety of cancers.

## Personalized Nutrition, Genetic Variants, and CRC

As mentioned above, diet can play a fundamental role in preventing or enhancing inflammatory diseases through different mechanisms. Dietary regimens characterized by a low caloric intake (e.g. Calorie restriction-CR or periodic fasting-PF) show several benefits in the prevention or treatment of human diseases. These eating patterns can act on several levels, such as strong anti-inflammatory action, for example in reducing the production of monocyte-derived interleukin-1 beta (IL-1β). Moreover, they can influence the microbiota composition with the reduction of pro-inflammatory strains and enrich the gut with probiotic-friendly strains. Lastly, nutritional patterns low in carbohydrates and proteins, among others CR and PF, can shift cellular metabolism from glycolysis to oxidative phosphorylation and act on nutrient-signaling pathways like IGF-1, PI3K, AKT-mTOR, and RAS-PKA, that activate stress resistance- genes and remodeling the epigenetic landscape regulating gene expression [91].

Personalized nutrition is based on individual’s information (e.g. age, insulin sensitivity, the composition of the gut microbiota) to enable the development of nutritional advice that patients can use to achieve a better lifestyle and long-lasting dietary behavior in a better way compared to that conventional, which comes from generic dietary advice. In the “omics” era, this approach has opened new avenues toward nutritional sciences, relying on the quantitative understanding of the association among individuals, phenotypes, and food intake [26].

The personalized nutritional approach offers several advantages including an individualized method with increased compliance, the potential for better health outcomes, and integration of advanced technologies. However, it has also several limitations like high costs and complexity, limited availability, still evolving science, and potential privacy concerns [72]. Nutritional therapies based on a personalized approach can be used against cancer, as an additional treatment, with well-established mechanisms of action including modulation of gene expression and regulation of signaling pathways implicated in key steps like proliferation, angiogenesis, metastasis or apoptosis. In this view, it has been demonstrated that nutritional strategies, through the inhibition of proliferative signaling as well as by stopping inflammation, can impact genetic instability associated with cancer [92].

Nowadays, in the case of CRC, some precision nutrition strategies are focused on lipid-metabolism-related genes. Lipids regulate several processes from ATP synthesis and the activation of essential cell-signaling pathways to membrane organization and plasticity. In this context, lipid-metabolism-related genes have acquired relevant interest since lipid metabolism can also impact several carcinogenic stages, either in primary neoplasia or metastasis [92]. In detail, the association between classical chemotherapeutic agents with bioactive compounds, which modulate lipid-metabolism-related gene expression, is one of the current lines of research in CRC treatment [93].

In recent years, it has been identified a number of nutritional compounds that have epigenetic targets in cancer cells and it has been introduced the term “epigenetic diet” to indicate commonly consumed bioactive dietary factors in some foods (soy, green tea, and cruciferous vegetables) which act to modify the epigenome and capable of protecting against cancer and the aging process [23].

**Fig. 2:**
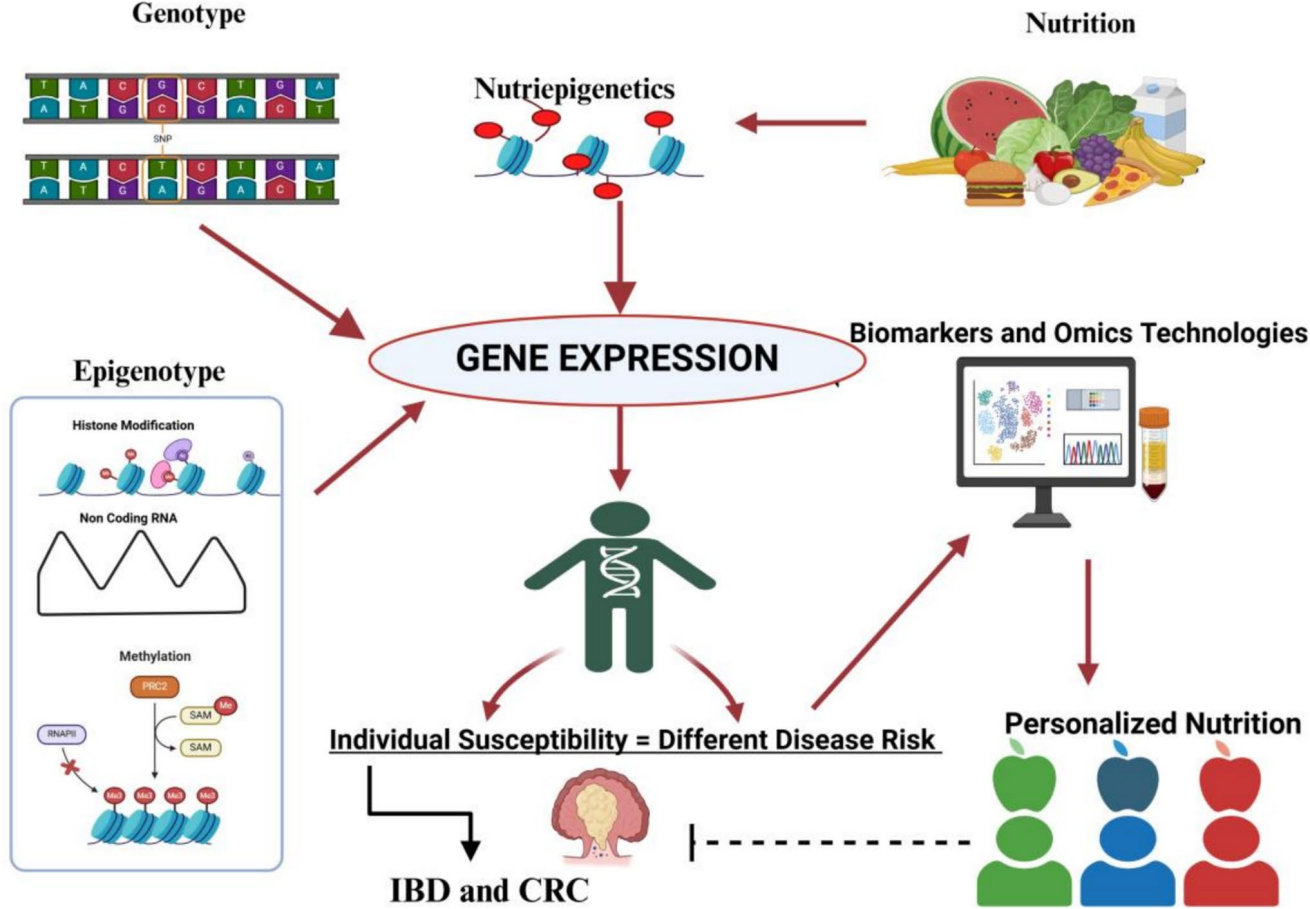
The complex interplay among genotype (SNPs), nutrition (Nutriepigenetics), and epigenotype (DNA methylation, histone modification, non-coding RNA). The interaction of these factors can increase the susceptibility to complex diseases including Inflammatory Bowel Diseases and Colorectal Cancer. The Omics technologies and the identification of specific biomarkers could help to detect the individual risk among people and therefore to develop specific nutrition personalized strategies. The original figure was created with BioRender.com

DNA methylation, which is the most investigated epigenetic biomarker, may be useful tool to detect the effects of environmental exposure. So, nutrition and dietary compounds can potentially affect the individual risk of diseases, in particular inflammatory diseases and cancer, with action on the genome through epigenetic modification [94] (Fig. 2). In this context, bioactive nutritional components of an epigenetic diet may be introduced in conjunction with one's regular dietary regimen and used therapeutically for medicinal or chemo preventive purposes [95].

After all, many dietary components can act influence phenotypic profile inducing epigenetic modification. For example, a diet low in fiber inhibits the production of the short-chain fatty acids (SCFAs: acetate, butyrate, and propionate) that are involved in the epigenetic control of the inflammatory process [96].

The alteration of DNA methylation is frequently observed in cancer. The status of several micronutrients (including folate, choline, betaine, and vitamins), which contribute to DNA methylation as methyl donors and co-factors, could offer potential preventive and therapeutic strategies in cancer.

The relation between folate and IBD is very complex: IBD patients show a lower levels of folate and the intestinal epithelial cells are affected by this nutritional deficit at the DNA methylation level. This could induce the dysregulation of genes involved in inflammation, immune response, and intestinal barrier function, thus influencing the pathogenesis of IBD. On the other hand, folate supplementation can do better results on the manifestation of IBD with a recovered gene expression pattern [28]. It has been demonstrated that folate also plays a role with dual action in CRC and higher levels of this element, also due to supplementation, are demonstrated to be beneficial in the prevention of colorectal cancer [97]. The change in blood levels of this vitamin is linked with DNA hypomethylation as well as hypermethylation of different tumor suppress genes; therefore subjects with the highest folate intake have a 30% reduction to develop CRC [98].

On the other hand, in the context of disease, once malignant transformation is already beginning, a low level of folate can be beneficial to stopping the progression of cell transformation [97]. Therefore, the relationship between folate intake and cancer is related to their capacity to reduce inflammatory markers, but, on the other side, the harmful effects of an excessive intake may also have adverse effects through epigenetic alterations [91].

One of the aims in the precision nutrition is the genotype-directed nutrition focused on gene variation and its consequences. Several SNPs in diet-nutrition-related genes are clearly associated with CRC [89]. Particularly, the most prevalent CRC is represented by sporadic CRC in which SNPs are more likely to be the cause rather than any other specific mutation [99]. A recent metanalysis demonstrated the correlation between the CRC risk with the genetic variants in the tumor suppressor gene *TP53* (rs1042522) and in the oncoprotein *MDM2* (rs2279744)*,* which downregulates the P53 pathway [100].

In addition, the methylenetetrahydrofolate reductase (*MTHFR)* gene is an example of an interaction between gene and nutrient, influencing serum levels of homocysteine mainly in subjects with a low dietary intake of folic acid [90]. Cys677Thr in *MTHFR* gene reduces enzymatic activity up to 30% [101]. This genetic variant and levels of folate intake are strictly related to regulate CRC risk, in fact, *TT* genotype is protective for CRC with high intake of folate while it represents a risk factor when low folate occurs [102]. Ferrari et al. [103], reported that folate intake, serum folate levels, global DNA methylation and patient age were predictors of clinicopathological staging of disease. Although the literature showed that folate is involved in DNA methylation, synthesis, and repair, as well as genetic variants in the *MTHFR* gene appear to be predictive of cancer treatment outcomes [104], the optimal folate levels are not critically identified. Further studies are need to evaluate if regular monitoring of folate may be clinically beneficial to improve cancer outcomes [105].

It’s well known that a diet rich in red and processed meat increases the risk of CRC up to 30%; due to cooking at high temperatures, producing harmful substances like heterocyclics amines (HCAs), PAHs and N-nitroso compounds(NOCs) [106]. These elements promotes DNA mutagenesis, and carcinogenesis, increasing CRC risk [107]. A genetic variant in the N-acetyltransferase (*NAT*) gene, implicated in the metabolic activation of heterocyclics amines, is strongly linked to his activity, and subjects with *GG* genotype of *NAT2* have a higher risk of CRC in relationship with a high consumption of meat [106]. Moreover, different polymorphisms in some genes like *COX-*2 (regulating the conversion of arachidonic acid into prostaglandins and the inflammatory response), the cytochrome P450 CYP enzymes (metabolization of carcinogenic compounds), and *NER* (restoring DNA damage) are linked to a higher risk of CRC in subjects with a bigger intake of meat.

In conclusion, there are different responses given by diverse intakes of nutrients in combination with genetic variants. Therefore, the general bits of advice for the population should be changed based on individual susceptibility based on the different genotypes [106].

There are other SNPs, linked to nutritional and metabolic factors, that influence CRC susceptibility; including variants in enzymes involved in detoxification like Glutathione S transferase or vitamin D receptors. Moreover, SNPs in genes related to the angiogenesis pathway (e.g. Vascular endothelial growth factor receptors FLT1 and KDR) lifestyle and environmental factors like smoking, dietary protein intake, and, alcohol consumption have been linked with CRC risk [93].

SMAD7 plays a role in the inhibition of the Transforming Growth Factor (TGF-ϐ) signaling pathway. TGF-ϐ pathway is involved in controlling several processes including cell proliferation; so the interaction between the *SMAD7* gene and the (TGF-ϐ) pathway could trigger the onset of cancer. rs4939827 in the SMAD7 gene was previously correlated with an increased risk of CRC. Alonso-Molero et al. investigated the relationship among the *SMAD7* rs4939827, the Mediterranean diet pattern, and the risk of colorectal cancer. The study demonstrated that the allele *C* in rs4939827, together with high adherence to the Mediterranean diet showed a protective effect on the onset of Colorectal carcinogenesis probably involving the TGF-ϐ pathway [108].

## Conclusion and Future Prospective

Cancer pathogenesis arises from complex interplay between genetic and environmental factors and among the latter dietary nutrients play an important role in cancer development. Moreover, the marked difference in cancer development with the same dietary intake among individuals could be due to the variation in their genetic variants, and this brings out the concept of nutrigenomics and nutrigenetics. To date, nutrigenomics is widely used not only for discussing the response to treatment of diet-related diseases but also for cancer pathogenesis [90]. In this view, the diet with its nutrients, through different actions, can indirectly (e.g. influencing weight and BMI, or through exposure to environmental pollutants ingested by foods) and directly (anti or pro-inflammatory activity, interaction with microbial gut composition, regulation of cellular metabolism pathways, interaction with epigenetic processes) play a key role in the prevention and management of the different gut diseases linked to inflammation, including CRC.

However, the increase of these diseases and the diagnosis of CRC in the younger population (age below 50) are becoming a public health issue. Therefore, the conventional nutritional recommendations developed to date are generic dietary advice related to food composition and dietary needs, without taking into account the specific cases, age groups, or modifiable lifestyle-related factors, that could trigger the pathogenetic process. In this context, integrating the analysis of risk factors in specific populations with the assessment of particular SNPs in individuals at major risk of the onset of CRC can result in personalized nutritional interventions based on individual susceptibility that can reverse the increasing trend of specific diseases, like CRC at an earlier age. In addition, since cancer is a multi-step process using many survival pathways to prevail over normal cells, it has been proposed the use of bioactive components, which have several molecular targets and suppressing multiple cellular pathways, could play a strong role in both prevention and treatment [89]. In detail, the bioactive nutrients and their metabolites, including tea polyphenols, genistein, curcumin, resveratrol, sulforaphane, and isothiocyanates, may serve more than a basic nutrition function and thus, could have a great potential in preventing cancer through modifying genetic and epigenetic targets.

Concerning this, potential novel DNA methylation biomarkers for the detection and diagnosis of cancer in the early stage could be useful to detect the amount of the individual’s environmental and food threat exposure that leads to the onset of specific diseases. This would allow acting on risk factors and individual behaviors to carry out specific nutrition strategies that can modify the epigenotype to decrease the risk of certain chronic diseases including cancer.

Moreover, with increasing knowledge of the function of bioactive food components and gut microbiota, it is likely that prebiotics and probiotics will play an important role in cancer prevention. In addition, gut microbiota can affect our epigenome. A newly emerging field of inquiry is related to small chemicals also called microbial metabolites, which are made by microbes. These small chemicals can be capable of altering host cell behavior, triggering epigenetic modifications thus, representing a potential way of preventing CRC [109]. Although our knowledge is yet limited, it has been suggested to introduce a strategy based on the selection of specific microbes and to develop engineered microbiota. The success of these approaches could add to the fast-growing functional nutraceutical industry [110], but future research directions in this dynamic field are needed.

Therefore, Nutriomics fields (including nutrigenomics, nutrigenetics, nutriepigenetics, nutrimetabolomics, and nutrimetagenomics) have emerged to understand the complex relationship between nutrients and the human body’s molecular processes, in order to, together with precision nutrition (dietary programs based on single characteristics), obtain better prevention and management of inflammatory chronic disease. These approaches should play an increasingly significant role, especially in the younger population which is nowadays more early exposed to food, and environmental insults to health than the people that were born before the 50 s as demonstrated from data of the onset of EoCRC. Nutrigenomics may proceed in respect of the effective management of cancer in an individualized nutritional consultation according to the individuals’ genetic profiles. Hence, utilizing these tools could be helpful to achieve better screening programs and consequently, a potential personalized nutritional approach to achieve optimal management of burden diseases like CRC in younger ones.

## Key References

- T. Hoang, S. Cho, J.-Y. Choi, D. Kang, and A. Shin, “Genome-Wide Interaction Study of Dietary Intake and Colorectal Cancer Risk in the UK Biobank,” *JAMA Netw. Open*, vol. 7, no. 2, p. e240465, Feb. 2024, 10.1001/jamanetworkopen.2024.0465. **Of outstanding importance**
  - In this article, genome-wide interaction analysis is performed to evaluate relationship between dietary factors and genetic variants. The risk of Colorectal Cancer (CRC) associated with fish intake is modulated by several variants in the *EPDR1* gene. In addition, potential pathways linking the consumption of milk, cheese, tea, and alcohol with CRC are suggested.
- E. K. Aglago et al*.*, “A Genetic Locus within the FMN1/GREM1 Gene Region Interacts with Body Mass Index in Colorectal Cancer Risk,” *Cancer Res.*, vol. 83, no. 15, pp. 2572–2583, Aug. 2023, 10.1158/0008-5472.CAN-22-3713**Of outstanding importance**
  - This article, through gene-environment interaction analysis, identifies a new locus in FMN1/GREM1 gene region interacting with BMI in the Colorectal Cancer Risk, suggesting potential implications for precision prevention strategies.
- K. Marangoni, G. Dorneles, D. M. Da Silva, L. P. Pinto, C. Rossoni, and S. A. Fernandes, “Diet as an epigenetic factor in inflammatory bowel disease,” World J. Gastroenterol., vol. 29, no. 41, pp. 5618–5629, Nov. 2023, 10.3748/wjg.v29.i41.5618. **Of importance**
  - The article emphasizes the role of diet, gut microbiota composition, and exercise in influencing epigenetic mechanisms and thereby affecting Inflammatory Bowel Disease (IBD) pathogenesis. It provides new insight into the future targeted approaches in the prevention and treatment of IBD.
- M. Franzago, L. Pilenzi, S. Di Rado, E. Vitacolonna, and L. Stuppia, “The epigenetic aging, obesity, and lifestyle,” Front. Cell Dev. Biol., vol. 10, p. 985274, Sep. 2022, 10.3389/fcell.2022.985274. **Of importance**
  - This article underlines the complex interaction between obesity and epigenetic aging on the chronic disease. Moreover, it focuses on the potential reversal of epigenetic modifications by a personalized diet- and lifestyle-based intervention.

## Data Availability

No datasets were generated or analysed during the current study.
